# High Fat Diet Increases Circulating Endocannabinoids Accompanied by Increased Synthesis Enzymes in Adipose Tissue

**DOI:** 10.3389/fphys.2018.01913

**Published:** 2019-01-10

**Authors:** Eline N. Kuipers, Vasudev Kantae, Boukje C. Eveleens Maarse, Susan M. van den Berg, Robin van Eenige, Kimberly J. Nahon, Anne Reifel-Miller, Tamer Coskun, Menno P. J. de Winther, Esther Lutgens, Sander Kooijman, Amy C. Harms, Thomas Hankemeier, Mario van der Stelt, Patrick C. N. Rensen, Mariëtte R. Boon

**Affiliations:** ^1^Department of Medicine, Division of Endocrinology, Leiden University Medical Center, Leiden, Netherlands; ^2^Einthoven Laboratory for Experimental Vascular Medicine, Leiden University Medical Center, Leiden, Netherlands; ^3^Division of Systems Biomedicine and Pharmacology, Leiden Academic Centre for Drug Research, Leiden University, Leiden, Netherlands; ^4^Department of Medical Biochemistry, Academic Medical Center, Amsterdam, Netherlands; ^5^Department of Diabetes/Endocrine, Lilly Research Laboratories, Lilly Corporate Center, Indianapolis, IN, United States; ^6^Institute for Cardiovascular Prevention (IPEK), Ludwig Maximilian University of Munich, Munich, Germany; ^7^Oxford Centre for Diabetes, Endocrinology and Metabolism, University of Oxford, Oxford, United Kingdom; ^8^Department of Molecular Physiology, Leiden Institute of Chemistry, Leiden University, Leiden, Netherlands

**Keywords:** brown adipose tissue, white adipose tissue, diet-induced obesity, endocannabinoids, NAPE-PLD

## Abstract

The endocannabinoid system (ECS) controls energy balance by regulating both energy intake and energy expenditure. Endocannabinoid levels are elevated in obesity suggesting a potential causal relationship. This study aimed to elucidate the rate of dysregulation of the ECS, and the metabolic organs involved, in diet-induced obesity. Eight groups of age-matched male C57Bl/6J mice were randomized to receive a chow diet (control) or receive a high fat diet (HFD, 45% of calories derived from fat) ranging from 1 day up to 18 weeks before euthanasia. Plasma levels of the endocannabinoids 2-arachidonoylglycerol (2-AG) and anandamide (N-arachidonoylethanolamine, AEA), and related *N*-acylethanolamines, were quantified by UPLC-MS/MS and gene expression of components of the ECS was determined in liver, muscle, white adipose tissue (WAT) and brown adipose tissue (BAT) during the course of diet-induced obesity development. HFD feeding gradually increased 2-AG (+132% within 4 weeks, *P* < 0.05), accompanied by upregulated expression of its synthesizing enzymes *Daglα* and β in WAT and BAT. HFD also rapidly increased AEA (+81% within 1 week, *P* < 0.01), accompanied by increased expression of its synthesizing enzyme *Nape-pld*, specifically in BAT. Interestingly, *Nape-pld* expression in BAT correlated with plasma AEA levels (*R*^2^ = 0.171, β = 0.276, *P* < 0.001). We conclude that a HFD rapidly activates adipose tissue depots to increase the synthesis pathways of endocannabinoids that may aggravate the development of HFD-induced obesity.

## Introduction

Obesity is becoming a global epidemic and the need for development of novel therapeutic interventions is high. The endocannabinoid system (ECS) is regarded as a potential therapeutic target since it regulates energy balance by influencing appetite (Foltin et al., 1988; Jamshidi and Taylor, 2001), intracellular lipolysis and energy expenditure [reviewed in (Cota, 2007; Mazier et al., 2015)]. The ECS consists of cannabinoid receptors, their endogenous ligands, the endocannabinoids, and the enzymes that synthesize and degrade the endocannabinoids. The cannabinoid receptors are G-protein-coupled receptors, comprising the CB1R and CB2R. The CB1R is expressed centrally and in peripheral metabolic tissues including white adipose tissue (WAT), brown adipose tissue (BAT), liver, skeletal muscle and the pancreas. In contrast, the CB2R is mainly expressed in immune cells (Howlett et al., 2002).

The two main circulating endocannabinoids are anandamide (*N*-arachidonoylethanolamine, AEA) and 2-arachidonoylglycerol (2-AG). Although they are both derived from cell membrane arachidonic acid (AA) derivatives, the levels of these endocannabinoids are differentially regulated. AEA can be generated via hydrolysis of *N*-acyl-phosphatidylethanolamines (NAPE) by a NAPE-specific phospholipase D (NAPE-PLD) and its degradation is primarily regulated by fatty acid amide hydrolase (FAAH). 2-AG levels are regulated by the biosynthesis enzymes diacylglycerol lipase α and β (DAGL-α and β) and the degradation enzyme mono-acylglycerol lipase (MAGL) (Muccioli, 2010; Bisogno and Maccarrone, 2014; Baggelaar et al., 2018).

Activation of the CB1R in peripheral tissues inhibits fatty acid oxidation resulting in a positive energy balance and thus development of obesity in mice (Ravinet Trillou et al., 2003; Osei-Hyiaman et al., 2005; Arrabal et al., 2015). In addition, an increased tone of the ECS is associated with obesity in humans (Engeli et al., 2005; Bluher et al., 2006). Efforts have been made to reverse obesity by blocking the CB1R by small molecules such as the inverse agonist rimonabant (Sam et al., 2011). Albeit that rimonabant was effective in humans as evident from sustained weight loss and reduction of dyslipidaemia, centrally mediated side effects resulted in removal from the market (Despres et al., 2005; Pi-Sunyer et al., 2006). Nevertheless, blocking the actions of the ECS is still regarded a potent therapeutic strategy [reviewed in (O’Keefe et al., 2014; Simon and Cota, 2017)].

To develop novel therapeutics that target specific aspects of the ECS, it is crucial to obtain more insight in how fast and in which organs the dysregulation of the ECS sets off. In this study, we aimed at elucidating these questions by exposing mice to a high fat diet (HFD) ranging from 1 day up to 18 weeks, which finally causes diet-induced obesity (DIO). We analyzed endocannabinoid levels in plasma as well as the expression of enzymes involved in endocannabinoid synthesis and breakdown in several metabolic organs.

## Materials and Methods

### Animals and Diet

Eighty-six 7-week old male C57B1/6J mice (Charles River Laboratories, United States) were obtained. All mice were group housed (3–4 mice per cage) under a 12 h:12 h light-dark cycle with *ad libitum* access to food and water. During the course of the experiment 7 out of 8 groups of mice were switched from a regular chow diet (Special Diets Services, United Kingdom) to a HFD (45% kcal fat, 35% kcal carbohydrate, 20% kcal protein, Special Diets Services, United Kingdom) in such a way that all mice were 25 weeks of age at the time of euthanasia. In total, the study consisted of eight groups receiving HFD for 0 day (control group, remaining on a regular chow diet), 1 day, 3 days, 1, 2, 4, 10, and 18 weeks (*n* = 10–11 per group). At the end of the study, mice were fasted overnight and subsequently euthanized by an injection with 0.25 mg ketamine and 0.05 mg xylazine per gram body weight. Blood was collected via a cardiac puncture with EDTA filled syringes and several organs (liver, quadriceps muscle, gonadal WAT and interscapular BAT) were isolated. The organs were immediately snap frozen in liquid nitrogen and stored at -80°C until further analysis. For BAT, a small piece was fixated for histological analysis. A second experiment was performed in which 14-week old male C57Bl/6J mice (Charles River Laboratories, United States) were fed 0 day or 1 week HFD (*n* = 8 per group) prior to euthanasia. Mice were fasted for 4 h and euthanized by CO_2_ suffocation. Blood was collected via cardiac puncture with EDTA filled syringes as described above. These studies were carried out in accordance with the recommendations of the animal experimentation guidelines of Amsterdam Medical Center (*n* = 86 experiment) and Leiden University Medical Center (*n* = 16 experiment) and approved by the local ethical review boards on animal experimentation.

### Endocannabinoid Plasma Levels

A liquid-liquid extraction using methyl tert-butyl ether as an organic solvent was used to extract endocannabinoids from plasma. Levels of endocannabinoids (AEA, 2-AG), *N*-acylethanolamines (NAEs), and AA were measured by UPLC-MS/MS (AB Sciex 6500 QTRAP) in 25 μL plasma samples. From the pool of individual study samples, quality controls (QCs) were used to generate calibration curves. Additionally, all samples were randomized and each batch of study samples included calibration samples, an even distribution of QC samples and blanks. The sample extraction procedure and method has been described in detail previously (Kantae et al., 2017).

### RNA Isolation and RT-PCR Analysis

RNA of liver and muscle was isolated using TriPure Isolation reagent (Roche, Netherlands) and 1 μg of RNA was reverse transcribed using Moloney Murine Leukemia Virus Reverse Transcriptase (Promega, Netherlands). For WAT and BAT, RNA was isolated using Trizol (Invitrogen, United States) and cDNA was synthesized using an iScript cDNA synthesis kit (Bio-Rad, Netherlands). RT-PCR was carried out on a CFX96 PCR machine (Bio-Rad) using IQ SYBR-Green Supermix (Promega). mRNA expression was normalized to *Hprt* and *36b4* as household genes for liver, BAT and WAT; for muscle mRNA expression was normalized to the expression of *36b4* only. Changes in gene expression relative to basal expression levels were only calculated if the average expression of at least the control group were Ct < 32. Primer sequences are listed in Table 1.

**Table 1:** List of primer sequences for RT-PCR.

Gene	Forward primer	Reverse primer
*Abhd4*	ATCCTCCAGTGTCTCCAGAACAA	GGGTCCCTTGGGAATGTTGG
*Cd68*	ATCCCCACCTGTCTCTCTCA	TTGCATTTCCACAGCAGAAG
*Daglα*	TATCTTCCTCTTCCTGCT	CCATTTCGGCAATCATAC
*Daglβ*	GGGTCTTTTGAGCTGTTC	AAGGAGGACTATCAGGTA
*Faah*	CAGCTACAAGGGCCATGCT	TTCCACGGGTTCATGGTCTG
*Gde1*	AAGGATTTTGTCTCCCCGGAC	ATGTAGCTGGACCCAAGGTG
*Hprt*	TTGCTCGAGATGTCATGAAGGA	AGCAGGTCAGCAAAGAACTTATAG
*Mgll*	CAGAGAGGCCAACCTACTTT	ATGCGCCCCAAGGTCATATTT
*Nape-pld*	AAAACATCTCCATCCCGAA	CGTCCATTTCCACCATCA
*Pla/at1*	CGGTAAATGATTGCTTCAGT	CCACAACATCCTTCAAAAGC
*Pla/at5*	CCTGGAGACCTGATTGAGA	GGTTGCTGAAGATAGAGGTG
*36b4*	GGACCCGAGAAGACCTCCTT	GCACATCACTCAGAATTTCAATGG


### Histology and Determination of Lipid Droplet Content in BAT

After dissection, a small piece of interscapular BAT was immediately fixated in 4% paraformaldehyde, subsequently dehydrated and embedded in paraffin. A Haematoxylin and Eosin staining was performed on paraffin sections using standard protocols. Intracellular lipid content was quantified with ImageJ (version 1.49).

### Statistical Analysis

All data are expressed as mean ± SEM. Data analysis was performed with an IBM SPSS Statistics 23 software package. Analysis between multiple groups was done by a one-way ANOVA with Dunnett’s *post hoc* test. For the analysis of plasma 2-AG in the second experiment we used an unpaired two-sided *t*-test. Furthermore, linear regression analysis computed by Pearson’s correlation was used to determine correlations. Significant differences are expressed relative to the chow-fed (0 week HFD) control group.

## Results

### HFD Feeding Rapidly Increases Plasma 2-AG and AEA Levels in Mice

To investigate the time course of the dysregulation of the ECS in the development of DIO, C57B1/6J mice were fed a HFD for 1 day up to 18 weeks. As expected, dietary intervention increased body weight (up to +51% after 18 weeks, *P* < 0.001, Table 2). Blood glucose levels rapidly increased in response to the HFD (+88% after 1 day of HFD, *P* < 0.001), whereas plasma TG levels increased more gradually (+155% after 2 weeks of HFD, *P* < 0.01) (Table 2).

**Table 2:** General characteristics of the mice during diet-induced obesity development.

	Duration of HFD (weeks), mean ± SEM							
	0	1/7	3/7	1	2	4	10	18
*N*=	11	11	10	10	11	11	11	11
Body weight (g)	27.3 ± 0.6	29.5 ± 0.6	31.1 ± 0.8∼	31.5 ± 0.5^∗^	34.4 ± 0.9^∗∗∗^	36.5 ± 1.2^∗∗∗^	38.1 ± 0.9^∗∗∗^	41.3 ± 2.0^∗∗∗^
Glucose (mg/dL)	69 ± 3	130 ± 15^∗∗∗^	118 ± 7^∗∗∗^	123 ± 4^∗∗∗^	104 ± 5^∗^	91 ± 4	112 ± 9^∗∗^	124 ± 7^∗∗∗^
Triglycerides (mM)	0.20 ± 0.02	0.25 ± 0.05	0.41 ± 0.09∼	0.34 ± 0.06	0.51 ± 0.07^∗∗^	0.81 ± 0.08^∗∗∗^	0.54 ± 0.03^∗∗^	0.60 ± 0.05^∗∗∗^


*Values are indicated in mean ± SEM. ∼P < 0.1, ^∗^P < 0.05, ^∗∗^P < 0.01, ^∗∗∗^P < 0.001 compared to the control group. These data were previously published elsewhere (van den Berg et al., 2016)*.

**FIGURE 1:**
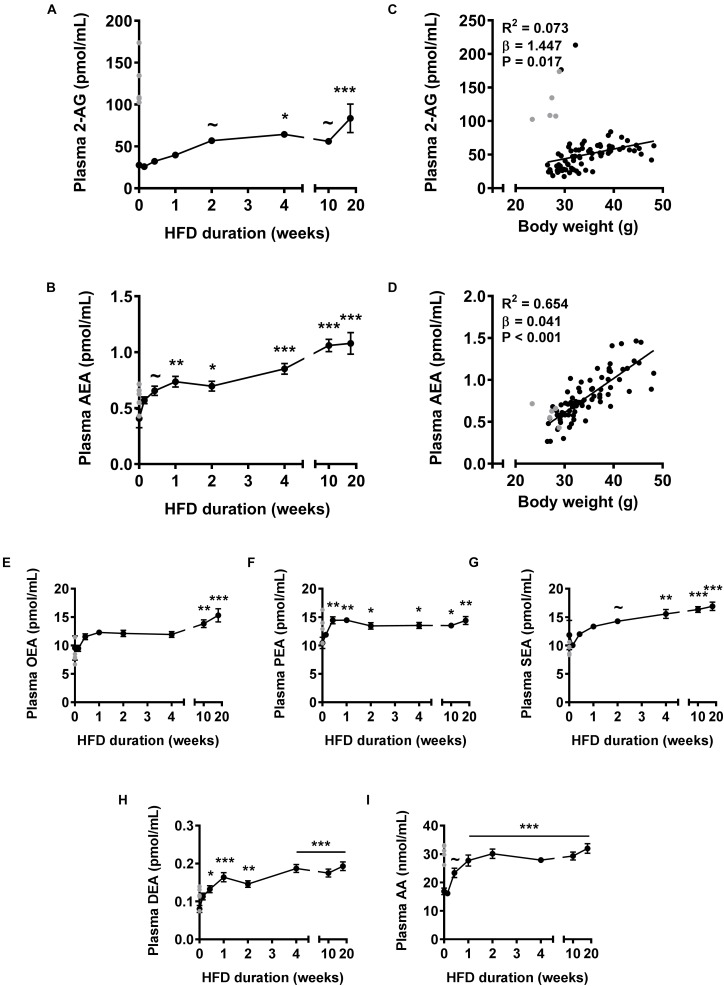
High fat diet feeding time-dependently increases plasma levels of 2-AG and AEA in mice. Liquid chromatography coupled with tandem mass spectrometry (LC-MS/MS) was used to determine plasma levels of 2-AG **(A)**, AEA **(B)**, OEA **(E)**, PEA **(F)**, SEA **(G)**, DEA **(H)**, and AA **(I)**. Data are mean ± SEM (*n* = 10–11). Error bars were too small to be visible for several data points (in **A,E–G,I**). ∼*P* < 0.1, ^∗^*P* < 0.05, ^∗∗^*P* < 0.01, ^∗∗∗^*P* < 0.001 compared to the control (0 week of HFD) group analyzed by one-way ANOVA with Dunnett’s *post hoc* test. In addition, linear regression analysis was performed on correlations between body weight and plasma levels of 2-AG **(C)** or AEA **(D)**, for all samples depicted in black (*n* = 81). Samples depicted in gray were regarded as biological outliers based on 2-AG and AA levels and therefore excluded from plasma data analyses.

First, we assessed plasma levels of the two main endocannabinoids in the course of DIO development (Figure 1). HFD feeding gradually increased 2-AG levels, which reached significance after 4 weeks (+132%, *P* < 0.05) and further increased up to 18 weeks (+201%; *P* < 0.001) (Figure 1A). Of note, in a few samples of the control group we observed extremely high 2-AG levels (>100 pmol/mL) that masked an initial increase in 2-AG levels upon HFD. To determine whether these high levels would represent (biological) outliers, we determined plasma 2-AG levels in a separate cohort of mice fed a HFD for 0 or 7 days. Indeed these values lie more than six standard deviations away from the average of the repeated control animals and could therefore be regarded as biological outliers. Importantly, we observed a trend toward elevated 2-AG plasma levels in the 7 days HFD fed group (+34%, *P* = 0.055, not shown) compared to the 0 day group in the repeated experiment. Therefore, we excluded the mice of the control group with extremely high plasma 2-AG levels from calculation of the means of all endocannabinoids and related metabolites, and instead indicated these data in gray (Figures 1A–I). HFD feeding also rapidly increased levels of AEA, the other main endocannabinoid, which reached significance after 1 week (+81%, *P* < 0.01) and further increased up to 18 weeks (+165%, *P* < 0.001) (Figure 1B). Next, we determined whether plasma endocannabinoid levels were related to body weight. Linear regression analysis on all data combined showed that body weight correlated weakly but positively with 2-AG levels (*R*^2^= 0.073, β = 1.447, *P* = 0.017, Figure 1C) and much more strongly with AEA levels (*R*^2^= 0.654, β = 0.041, *P* < 0.001, Figure 1D).

NAPE-PLD does not only produce AEA, but generates a whole family of *N*-acylethanolamines (NAEs), including N-oleoylethanolamine (OEA), N-palmitoylethanolamine (PEA), N-stearoylethanolamine (SEA), and *N*-docosatetraenoylethanolamine (DEA). Similar to AEA, plasma concentrations of these non-cannabinoid fatty acid amides were raised in response to HFD feeding, albeit with different kinetics (Figures 1E–H). Plasma levels of AA, the precursor and degradation product of 2-AG and AEA (Piomelli, 2003), also rapidly increased in the first 2 weeks of HFD after which a plateau was reached (+89% after 18 weeks, *P* < 0.001, Figure 1I). Of note, the samples of the control group with extremely high 2-AG levels (Figure 1A) also showed elevated AA levels (Figure 1I).

### HFD Feeding Increases Expression of 2-AG Synthesis and Degradation Enzymes in WAT and BAT

**FIGURE 2:**
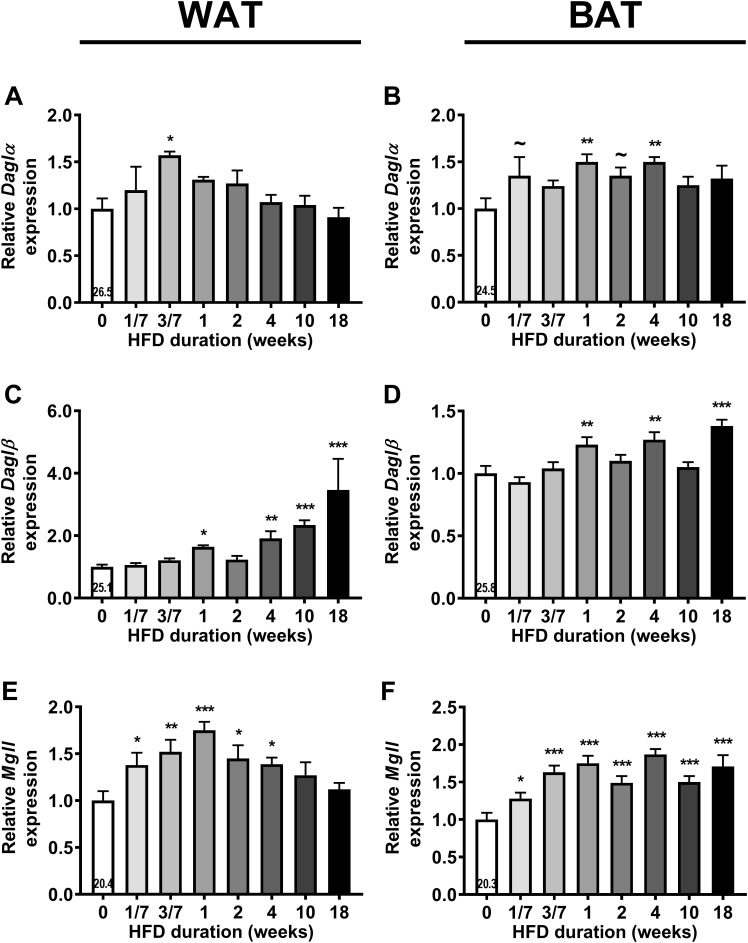
High fat diet (HFD) feeding increases the expression of 2-AG synthesis and degradation enzymes in WAT and BAT. Relative gene expression of 2-AG synthesis enzymes *Daglα* **(A,B)**, *Daglβ* **(C,D)**, and degradation enzyme *Mgll* **(E,F)** in WAT **(A,C,E)** and in BAT **(B,D,F)**. The number in the column of the 0 week of HFD group indicates the average CT value of that treatment group for that gene. Data are mean + upper SEM (*n* = 10–11) ∼*P* < 0.1, ^∗^*P* < 0.05, ^∗∗^*P* < 0.01, ^∗∗∗^*P* < 0.001 compared to the control (0 week of HFD) group analyzed by one-way ANOVA with Dunnett’s *post hoc* test.

Next, we assessed gene expression of the enzymes responsible for the synthesis (*Daglα* and *Daglβ*) and degradation (*Mgll*) of 2-AG in liver, muscle, WAT and BAT (Figure 2; an overview of all relative gene expressions of the enzymes involved in synthesis and degradation is shown in Supplementary Table S1). HFD feeding transiently increased *Daglα* expression in WAT after 3 days (+57%, *P* < 0.05, Figure 2A) and increased *Daglα* expression in BAT after 1 and 4 weeks of HFD (Figure 2B). HFD feeding also induced *Daglβ* expression in WAT (Figure 2C) and BAT (Figure 2D), especially toward the end of the intervention (i.e., +246% in WAT and +38% in BAT after 18 weeks). Linear regression analyses between the expression levels of DAG lipases in adipose tissues and plasma 2-AG levels showed inconclusive data (Supplementary Figure S1). HFD feeding also transiently increased *Mgll* expression in WAT reaching a peak after 1 week of HFD (+75%, *P* < 0.001), which normalized toward the end of HFD intervention (Figure 2E). In contrast, HFD induced a sustained increase in *Mgll* expression levels in BAT from 1 day on (+28%, *P* < 0.05, Figure 2F). HFD feeding did not persistently affect gene expression of synthesis and degradation enzymes in liver and muscle. It only temporarily increased *Daglα* expression in muscle (at 2 weeks), increased *Daglβ* expression (at 3 days) and decreased *Mgll* expression (at 1 day) in liver (data in Supplementary Table S1). Collectively, these data show that the rise in 2-AG levels during DIO development coincided with enhanced expression of synthesis and degradation enzymes specifically in WAT and BAT.

### HFD Feeding Increases *Nape-pld* Expression in WAT and BAT

**FIGURE 3:**
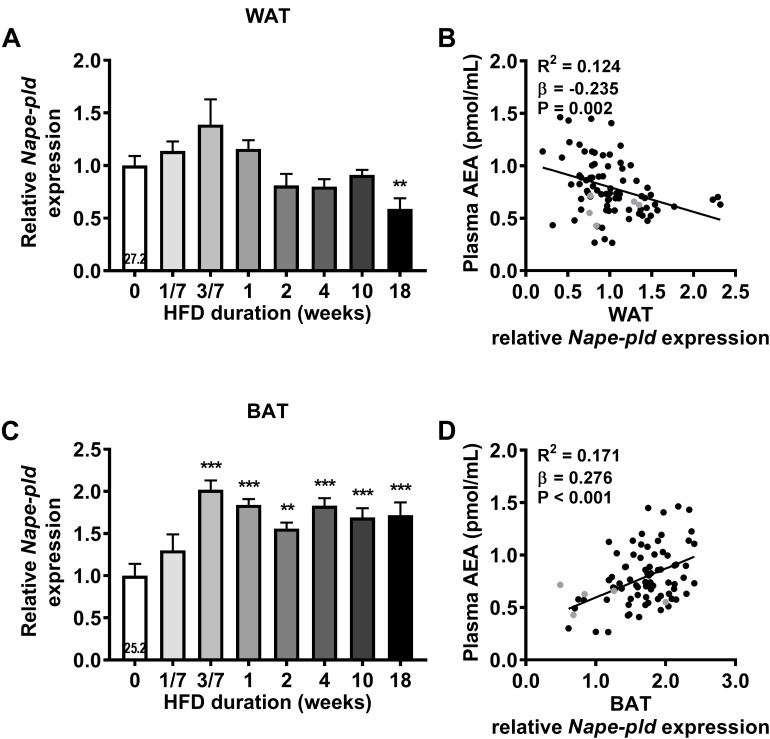
High fat diet feeding upregulates *Nape-pld* expression in WAT and in BAT. Relative gene expression of AEA synthesis enzyme *Nape-pld* in WAT **(A)**, BAT **(C)**. The number in the column of the 0 week of HFD group indicates the average CT value of that treatment group for that gene. Data are mean + upper SEM (*n* = 10–11). ^∗∗^*P* < 0.01, ^∗∗∗^*P* < 0.001 compared to the control (0 week of HFD) group analyzed by one-way ANOVA with Dunnett’s *post hoc* test. In addition, linear regression analysis was performed on correlations between *Nape-pld* expression relative to 0 week of HFD in WAT **(B)** or in BAT **(D)** and plasma levels of AEA, for all samples depicted in black (*n* = 81). Samples depicted in gray were regarded as biological outliers based on 2-AG and AA levels and therefore excluded from linear regression analyses.

We next assessed gene expression of the enzyme responsible for AEA and NAEs synthesis (*Nape-pld*) in the various metabolic organs. HFD feeding tended to increase *Nape-pld* expression in WAT after 3 days, although expression levels normalized thereafter and were decreased after 18 weeks (-41%, *P* < 0.01, Figure 3A). Surprisingly, linear regression analysis showed a small, but significant, negative correlation between *Nape-pld* expression in WAT and plasma AEA levels (*R*^2^= 0.124, β = -0.235, *P* = 0.002, Figure 3B). Interestingly, HFD feeding increased *Nape-pld* expression in BAT starting at 3 days (+102%, *P* < 0.001, Figure 3C), after which levels reached a plateau. Moreover, *Nape-pld* expression in BAT positively correlated with plasma AEA levels (*R*^2^ = 0.171, β = 0.276, *P* < 0.001, Figure 3D), supporting a contribution of BAT *Nape-pld* expression to circulating AEA levels. HFD feeding decreased the expression of *Nape-pld* in muscle reaching significance from 4 weeks onwards and did not affect *Nape-pld* expression in de liver (data in Supplementary Table S1). We also determined the potential contribution of the expression of genes involved in the phospholipase A/acyltransferase (PLA/AT) family, which can produce NAPE in a Ca^2+^-independent manner, in the increase in AEA levels in DIO (Hussain et al., 2017). However, expression of the PLA/AT (HRAS-like suppressor) gene family was either too low to detect (*Pla/at1* in liver, WAT, BAT and *Pla/at5* in liver) or did not show a clear or persistent rise in expression levels that could explain the rise in AEA levels (not shown). Alpha/beta hydrolase domain containing-4 (ABHD4) and glycerophosphodiesterase-1 (GDE1) have been suggested to be involved in AEA synthesis by BAT (Krott et al., 2016). However, time-dependent expression levels of *Abhd4* and *Gde1* in BAT did not coincide with the HFD-induced rise in AEA and NAEs (not shown).

Next, we determined gene expression levels of *Faah*, the enzyme involved in AEA and NAEs degradation. HFD feeding decreased *Faah* expression levels in the liver after 18 weeks (-41%, *P* < 0.01, Supplementary Table S1). Expression levels of *Faah* in muscle, WAT and BAT were too low to be detected. Altogether, these data show that HFD robustly increased the expression of *Nape-pld* in BAT, suggesting that this tissue may contribute to the increased plasma AEA and NAE levels during DIO development.

### Plasma AEA Levels Positively Correlate With Lipid Content of BAT

**FIGURE 4:**
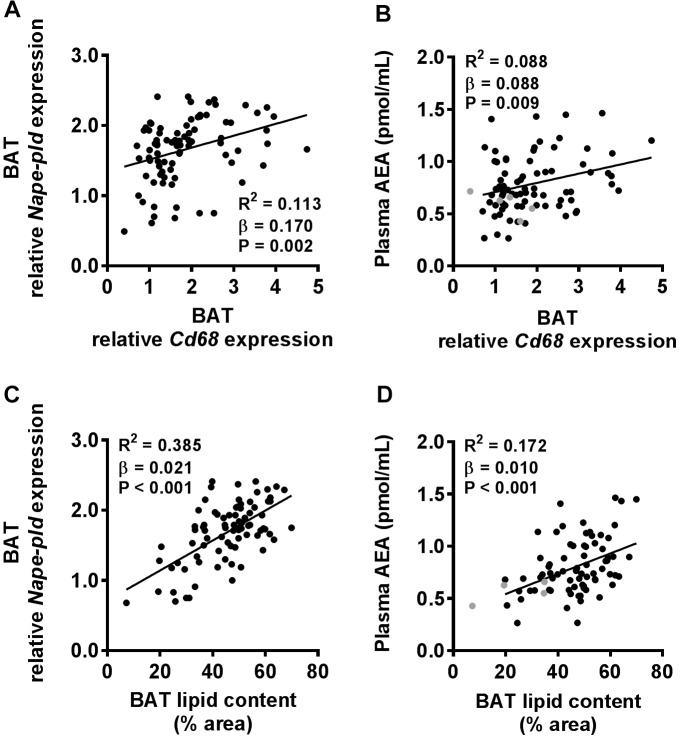
Both macrophage marker expression in BAT and lipid content of BAT positively correlate with plasma AEA levels. Linear regression analysis was performed on correlations between *Cd68* expression relative to 0 week of HFD in BAT and *Nape-pld* expression in BAT **(A)** or plasma levels of AEA **(B)**. Also, linear regression analysis was performed on correlations between lipid content of BAT and *Nape-pld* expression relative to 0 week of HFD in BAT **(C)** or plasma levels of AEA **(D)**. Correlations are shown for all samples depicted in black (*n* = 86 in **A,C** and *n* = 81 in **B,D**). Samples depicted in gray were regarded as biological outliers based on 2-AG and AA levels and therefore excluded from linear regression analyses.

Next, we aimed to gain more insight into the cell types within BAT that may have contributed to the robust increased *Nape-pld* expression and plasma AEA levels in DIO development. Because macrophages have been shown to produce AEA (Di Marzo et al., 1996), we first determined gene expression levels of the macrophage marker *Cd68* in BAT. Linear regression analysis showed a weak positive correlation between *Cd68* expression and *Nape-pld* expression in BAT (*R*^2^= 0.113, β = 0.170, *P* = 0.002, Figure 4A) and plasma AEA levels (*R*^2^ = 0.088, β = 0.088, *P* = 0.009, Figure 4B). Besides macrophages, brown adipocytes might also be involved. Since intracellular lipid droplets have been shown to co-localize with intracellular AEA (Oddi et al., 2008), we quantified BAT lipid droplet content in H&E stained BAT sections (Supplementary Figure S2). Compared to *Cd68*, BAT lipid droplet content showed a more pronounced positive correlation with *Nape-pld* expression levels in BAT (*R*^2^= 0.385, β = 0.021, *P* < 0.001, Figure 4C) as well as plasma AEA levels (*R*^2^= 0.172, β = 0.010, *P* < 0.001, Figure 4D).

## Discussion

In this study, we demonstrated that HFD feeding increases circulating levels of endocannabinoids, with a rapid initial increase in AEA and a more gradual increase in 2-AG, in the course of DIO development. These changes were accompanied by increased gene expression of the synthesis and degradation enzymes of 2-AG in both WAT and BAT, and with increased expression of the AEA synthesis enzyme *Nape-pld* in BAT. Taken together, these data indicate that the dysregulation of the ECS in the development of obesity occurs rapidly and that WAT and BAT might contribute to these effects.

The observed increases in endocannabinoids in the development of DIO are in agreement with previous studies in mice that showed increased plasma 2-AG and AEA levels after 9 weeks (D’Eon et al., 2008) and 36 weeks (Pati et al., 2018) of HFD feeding and increased plasma AEA levels in a model for glucocorticoid induced obesity (Bowles et al., 2015). These data also are concordant with data in humans, since obese individuals have higher 2-AG levels compared to lean individuals (Engeli et al., 2005; Bluher et al., 2006; Cote et al., 2007). It was somewhat surprising that a subset of the control group that were not fed a HFD showed very high 2-AG levels in addition to AA levels, the reason of which is currently unclear to us. By performing a second study in mice we confirmed that HFD feeding induced an initial rise rather than decrease in 2-AG levels. Thus, although the reason for the very high 2-AG levels in a subgroup is intriguing, we regarded those mice as biological outliers. Besides 2-AG and AEA, HFD feeding also increased the plasma levels of AA. Since AA is constituent and degradation product of 2-AG and AEA, elevated AA levels may either be a cause or consequence of the increased levels. HFD feeding also increased plasma levels of other *N*-acylethanolamines, including OEA, PEA, SEA and DEA. These NAEs have other biological targets involved in controlling the energy balance, such as peroxisome proliferator-activated receptors-α (PPARα), PPARγ and G protein-coupled receptor 119 (Fezza et al., 2014).

Currently, it is unknown which organs contribute to the increased plasma endocannabinoid levels in HFD-induced obesity (Hillard, 2018). We showed that body weight positively correlates with plasma endocannabinoid levels, albeit that the correlation with AEA (*R*^2^= 0.654) is stronger than with 2-AG (*R*^2^= 0.073). Since body weight differences in the range of approximately 30–50 g, as observed in this study, are mainly caused by differences in body fat (van Beek et al., 2015), it was considered likely that engulfment of lipids by adipocytes and/or expansion of the adipocyte pool would contribute to the increase in endocannabinoids. Insulin resistance, which is closely linked to increased intracellular lipid deposition (Tchernof and Despres, 2013) is also associated with a dysregulated ECS (Gruden et al., 2016). By performing gene expression analysis in metabolically active organs, we could demonstrate that expression of enzymes involved in endocannabinoid synthesis increased in WAT as well as BAT. This is in full agreement with a previous study in which 3 and 8 weeks of HFD feeding, with a diet closely resembling the HFD used in our experiments, resulted in increased local levels of endocannabinoids (AEA and 2-AG) in BAT (Matias et al., 2008). The increase in plasma 2-AG coincided with increased gene expression of DAGLα and DAGLβ in both WAT and BAT. Given the different time-course of expression, where the increase in *Daglα* seems to precede the increase in *Daglβ*, we postulate that DAGLα may be responsible for the initial rise in 2-AG, while DAGLβ may mediate the late increase in 2-AG. Similarly, the increase in plasma AEA coincided with increased gene expression of its synthesizing enzyme NAPE-PLD in BAT. Moreover, plasma AEA correlated positively with *Nape-pld* expression in BAT but not in WAT. It is therefore likely that BAT rather than WAT contributes to the rise in AEA levels. In this respect, it is interesting that expression of *Nape-pld* is higher in BAT than in WAT, as evident from lower Ct values. The expression levels of most of the enzymes showed a sharp increase in the first week of HFD feeding, which coincided with the timing of the largest increase in lipid deposition in BAT. In BAT, ABHD4, and GDE1 were shown to also be involved in AEA synthesis and their expression respond to BAT activating agents (Krott et al., 2016), although we did not find the expression levels of these enzymes to coincide with the increase in circulating AEA and NAEs levels with prolonged HFD feeding. Of note, expression of the endocannabinoid degradation enzymes *Faah* and *Mgll* in the adipose tissues were either undetectable or increased. Although we have not been able to measure actual enzyme activities due to technical reasons, it is tempting to speculate that net whole body endocannabinoid synthesis exceeds degradation since circulating levels increase in the course of DIO. Synthesis enzymes of 2-AG in liver and skeletal muscle were only transiently increased, and the synthesis enzyme of AEA was decreased in skeletal muscle. Thus, although we cannot exclude the contribution of other organs as source for plasma endocannabinoid levels (e.g., brain and intestine), our data suggest that WAT and BAT are likely important organs that release 2-AG and AEA levels in HFD-induced obesity.

It is interesting to speculate on the cellular source within the adipose tissue depots that is involved in endocannabinoid synthesis. HFD-induced development of DIO causes accumulation of macrophages in WAT (van Beek et al., 2015) as well as BAT (Van den Berg S. M., unpublished). Macrophages are able to produce AEA (Di Marzo et al., 1996), and we found a positive correlation between macrophage marker *Cd68* and *Nape-pld* expression in BAT as well as with AEA levels in plasma. However, the concentration of macrophages in adipose tissue is relatively low, even in obesity, and stronger positive correlations were found between the lipid content in BAT and both *Nape-pld* expression and plasma AEA levels. Therefore, adipocytes likely contribute substantially more to the circulating endocannabinoid pool than macrophages. This hypothesis is corroborated by previous findings that AEA co-localizes with adiposomes or lipid droplets *in vitro* (Oddi et al., 2008) and that specific deletion of NAPE-PLD in adipocytes of mice decreased levels of PEA, OEA and SEA in WAT, despite increased inflammation and influx of macrophages (Geurts et al., 2015). In our study, HFD feeding increases the cellular mRNA levels of the synthesizing enzymes in adipose tissue. In addition, expansion of the total number of adipocytes in the time course of DIO further increases whole body expression of these enzymes. The rapid increases in gene expression in BAT may be explained by the rapid whitening of BAT as induced by HFD feeding (Shimizu et al., 2014). Indeed, BAT lipid droplet content positively correlated with *Nape-pld* expression and AEA plasma levels. Furthermore, these data are in line with the recent observation that acute activation of BAT decreases *Nape-pld* expression (Krott et al., 2016). Interestingly, the concentration of 2-AG and AEA is higher in BAT than WAT (Krott et al., 2016), suggesting at least a role of BAT in determining circulating endocannabinoid levels. Collectively, it is likely that lipid-filled adipocytes rather than macrophages within the adipose tissues contribute to circulating plasma endocannabinoid levels.

In our study, we found no evidence for a contribution of decreased degradation pathways of 2-AG and AEA in adipose tissues determining plasma levels of endocannabinoids. Specifically, we were unable to detect any expression of AEA degradation enzyme *Faah* in WAT, BAT and muscle, and found decreased liver *Faah* expression after 18 weeks of HFD feeding. This is in line with the fact that FAAH was reported to play an important role in obesity. Notably, a missense polymorphism in the FAAH gene is associated with obesity in humans (Sipe et al., 2005) and FAAH deficient mice have increased AEA levels in, e.g., the liver and show increased fat mass and body weight (Tourino et al., 2010). On the other hand, Bartelt et al. (2011) found that HFD feeding for 16 weeks in mice caused a decrease in *Faah* expression and FAAH enzymatic activity in WAT which was accompanied by increased AEA in this tissue. To what extend catabolism of AEA and 2-AG by adipose tissue determines circulating levels of these endocannabinoids warrants further study.

It is tempting to speculate on the biological role of the increases of 2-AG and AEA in the time course of HFD-induced obesity. Endocannabinoids are known to decrease insulin sensitivity (Gruden et al., 2016) and to reduce sympathetic responses by inhibiting noradrenergic signaling (Quarta et al., 2011; Krott et al., 2016), and thereby decrease lipolysis in WAT and thermogenesis in BAT. Possibly, in case of acute lipid overload, as mimicked by a switch from regular chow to a HFD, initial accumulation of lipids in WAT and BAT drives the synthesis pathways of endocannabinoids that can have autocrine and even paracrine effects on these organs to inhibit sympathetic signaling. This sequence of events reduces intracellular lipolysis in BAT and WAT, thereby resulting in reduced thermogenesis in BAT and increased triglyceride storage in WAT. Such as a feed-forward mechanism may thus allow the body to store excess lipids effectively in adipose tissues. Interestingly, we have shown that inhibition of endocannabinoid signaling by strictly peripheral CB1R antagonism activates BAT and reduces adiposity in HFD-fed mice (Boon et al., 2014). In theory, HFD induced lipid accumulation can lead to increased endocannabinoid synthesis which attenuates BAT and WAT activity and results in a positive energy balance.

## Conclusion

The time course of HFD-induced obesity plasma endocannabinoid levels rapidly rise as most probably explained by increased synthesis pathways in adipose tissue depots. We speculate that this sequence of events may attenuate sympathetic signaling in these tissues by CB1R agonism, which would result in reduced thermogenesis and increased storage of excess lipids in WAT. Given the fact that strictly peripheral CB1R antagonism activates BAT and reduces adiposity in mice, we anticipate that strategies inhibiting CB1R selectively on (brown) adipocytes or reducing endocannabinoid synthesis by adipocytes may be a worthwhile strategy to pursue in combating obesity and associated disorders.

## Author Contributions

EK and VK performed the experiments, analyzed the data, wrote the manuscript, and contributed to the discussion. VK developed and validated the UPLC-MS/MS method to quantify endocannabinoids, NAEs and AA in murine plasma. BM and RvE analyzed the data, contributed to the discussion, and reviewed/edited the manuscript. SvdB designed the study, performed the *in vivo* experiment and kindly provided the samples for analysis, and reviewed and edited the manuscript. MdW and EL designed the study and reviewed and edited the manuscript. KN, SK, AH, and TH contributed to the discussion and reviewed and edited the manuscript. AR-M and TC reviewed and edited the manuscript. MvdS, PR, and MB designed and supervised the project, contributed to the discussion and reviewed and edited the manuscript.

## Conflict of Interest Statement

AR-M and TC are employees of Lilly. The remaining authors declare that the research was conducted in the absence of any commercial or financial relationships that could be construed as a potential conflict of interest.

## Abbreviations

2-AG: 2-arachidonoylglycerol
AA: arachidonic acid
ABHD4: *Abhd4* Alpha/beta hydrolase domain containing-4
AEA: anandamide (*N*-arachidonoylethanolamine)
BAT: brown adipose tissue
CB1R: cannabinoid receptor type 1
CB2R: cannabinoid receptor type 2
DAGL-α/β: *Dagl-α/β* diacylglycerol lipase-α/β
DEA: *N*-docosatetraenoylethanolamine
DIO: diet-induced obesity
ECS: endocannabinoid system
FAAH: *Faah* fatty acid amide hydrolase
GDE1: *Gde1* glycerophosphodiesterase-1
gWAT: gonadal white adipose tissue
HFD: high fat diet
LC-MS/MS: liquid chromatography coupled tandem mass spectrometry
MAGL: *Mgll* monoacylglycerol lipase
NAE: *N*-acylethanolamine
NAPE: *N*-acylphosphatidylethanolamine
NAPE-PLD: *Nape-pld N*-acylphosphatidylethanolamine phospholipase D
OEA: *N*-oleoylethanolamine
PEA: *N*-palmitoylethanolamine
SEA: *N*-stearoylethanolamine
