# Supplementary material for: High Fat Diet Increases Circulating Endocannabinoids Accompanied by Increased Synthesis Enzymes in Adipose Tissue
Source: Front Physiol. 2019 Jan 10;9:1913. doi: 10.3389/fphys.2018.01913 (PMC6335353; doi:10.3389/fphys.2018.01913)
Supplement: Supplementary file 1 [file Data_Sheet_1.docx]

|  | | HFD intervention (weeks) | | | | | | | |  |
| --- | --- | --- | --- | --- | --- | --- | --- | --- | --- | --- |
|  |  | **0** | **1/7** | **3/7** | **1** | **2** | **4** | **10** | **18** | ***P*-value** |
| Liver | *Daglα* | 1.00  (0.93, 1.08) | 0.93  (0.86, 1.00) | 1.16  (1.05, 1.29) | 1.16  (0.99, 1.34) | 0.96  (0.85, 1.09) | 1.24  (1.14, 1.35) | 1.14  (1.05, 1.23) | 1.20  (1.14, 1.27) | 0.266 |
|  | *Daglβ* | 1.00  (0.96, 1.05) | 1.09  (1.04, 1.15) | 1.21*  (1.17, 1.25) | 0.96  (0.93, 0.98) | 0.97  (0.93, 1.01) | 0.95  (0.91, 0.99) | 0.96  (0.90, 1.03) | 0.93  (0.90, 0.96) | 0.001 |
|  | *Mgll* | 1.00  (0.93, 1.08) | 0.65***  (0.59, 0.71) | 1.03  (0.96, 1.10) | 0.79  (0.79, 0.80) | 1.05  (1.00, 1.10) | 0.99  (0.94, 1.05) | 0.91  (0.86, 0.98) | 0.86  (0.80, 0.92) | <0.001 |
|  | *Nape-pld* | 1.00  (0.89, 1.12) | 0.92  (0.81, 1.06) | 1.16  (1.06, 1.27) | 1.12  (1.04, 1.22) | 1.21  (1.06, 1.37) | 1.09  (1.00, 1.19) | 1.20  (1.10, 1.30) | 1.11  (0.88, 1.40) | 0.792 |
|  | *Faah* | 1.00  (0.94, 1.06) | 1.10  (1.04, 1.16) | 1.30  (1.21, 1.40) | 0.93  (0.82, 1.06) | 1.20  (1.12, 1.28) | 0.87  (0.77, 0.99) | 0.94  (0.82, 1.08) | 0.59**  (0.49, 0.70) | <0.001 |
| Muscle | *Daglα* | 1.00  (0.76, 1.32) | 1.41  (1.16, 1.71) | 1.43  (1.32, 1.56) | 1.45  (1.38, 1.53) | 1.82**  (1.72, 1.93) | 1.39  (1.30, 1.49) | 1.47  (1.40, 1.53) | 1.00  (0.89, 1.14) | 0.009 |
|  | *Daglβ* | 1.00  (0.86, 1.16) | 0.95  (0.84, 1.08) | 1.03  (1.00, 1.07) | 1.02  (0.99, 1.05) | 1.24  (1.17, 1.30) | 1.15  (1.10, 1.20) | 1.14  (1.09, 1.20) | 0.97  (0.92, 1.01) | 0.073 |
|  | *Mgll* | 1.00  (0.76, 1.32) | 0.79  (0.66, 0.95) | 1.48  (1.21, 1.81) | 1.06  (0.91, 1.23) | 1.45  (1.27, 1.66) | 1.24,  (1.10, 1.40) | 1.20  (1.10, 1.30) | 0.89  (0.79, 1.00) | 0.060 |
|  | *Nape-pld* | 1.00  (0.91, 1.09) | 0.72  (0.61, 0.85) | 0.86  (0.75, 0.98) | 0.71  (0.65, 0.78) | 0.88  (0.81, 0.97) | 0.58**  (0.55, 0.60) | 0.67~  (0.64, 0.71) | 0.62*  (0.55, 0.71) | 0.010 |
|  | *Faah* | ND |  |  |  |  |  |  |  |  |
| WAT | *Daglα* | 1.00  (0.90, 1.11) | 1.20  (0.99, 1.45) | 1.57*  (1.53, 1.61) | 1.31  (1.28, 1.34) | 1.27  (1.14, 1.41) | 1.07  (1.00, 1.15) | 1.04  (0.95, 1.14) | 0.91  (0.83, 1.01) | 0.028 |
|  | *Daglβ* | 1.00  (0.93, 1.07) | 1.06  (1.00, 1.12) | 1.21  (1.15, 1.27) | 1.64*  (1.59, 1.69) | 1.23  (1.12, 1.35) | 1.91**  (1.70, 2.14) | 2.34***  (2.19, 2.49) | 3.46***  (2.68, 4.46) | <0.001 |
|  | *Mgll* | 1.00  (0.91, 1.10) | 1.38*  (1.26, 1.51) | 1.52**  (1.41, 1.65) | 1.75***  (1.67, 1.84) | 1.45*  (1.33, 1.59) | 1.39*  (1.32, 1.46) | 1.27  (1.14, 1.41) | 1.12  (1.06, 1.19) | <0.001 |
|  | *Nape-pld* | 1.00  (0.92, 1.09) | 1.14  (1.05, 1.23) | 1.39  (1.18, 1.63) | 1.16  (1.08, 1.24) | 0.81  (0.72, 0.92) | 0.80  (0.73, 0.87) | 0.91  (0.87, 0.96) | 0.59**  (0.51, 0.69) | <0.001 |
|  | *Faah* | ND |  |  |  |  |  |  |  |  |
| BAT | *Daglα* | 1.00  (0.90, 1.11) | 1.35~  (1.17, 1.55) | 1.24  (1.19, 1.30) | 1.50**  (1.43, 1.58) | 1.35~  (1.27, 1.44) | 1.50**  (1.45, 1.55) | 1.25  (1.17, 1.34) | 1.32  (1.19, 1.46) | 0.030 |
|  | *Daglβ* | 1.00  (0.95, 1.06) | 0.93  (0.90, 0.97) | 1.04  (1.00, 1.09) | 1.23**  (1.17, 1.29) | 1.10  (1.05, 1.15) | 1.27**  (1.22, 1.33) | 1.05  (1.00, 1.09) | 1.38***  (1.33, 1.43) | <0.001 |
|  | *Mgll* | 1.00  (0.92, 1.09) | 1.28*  (1.20, 1.36) | 1.63***  (1.54, 1.72) | 1.75***  (1.66, 1.85) | 1.49***  (1.42, 1.58) | 1.87***  (1.81, 1.94) | 1.50***  (1.43, 1.58) | 1.71***  (1.57, 1.86) | <0.001 |
|  | *Nape-pld* | 1.00  (0.88, 1.14) | 1.30  (1.14, 1.49) | 2.02***  (1.92, 2.13) | 1.84***  (1.77, 1.91) | 1.56**  (1.49, 1.63) | 1.83***  (1.74, 1.92) | 1.69***  (1.59, 1.80) | 1.72***  (1.59, 1.87) | <0.001 |
|  | *Faah* | ND |  |  |  |  |  |  |  |  |

**Supplementary Table 1.** Relative gene expression levels of synthesis and degradation enzymes *Daglα*, *Daglβ*, *Mgll* (of the 2-AG pathway) and *Nape-pld* and *Faah* (of the AEA pathway). Data are mean (with lower, upper SEM) (n=10-11). ND (not detected) indicates the average CT values were >32. ~*P*<0.1; **P*<0.05; ***P*<0.01; ****P*<0.001 compared to the control (0 weeks of HFD) group analysed by one-way ANOVA with Dunnett’s posthoc test.
