# Supplementary material for: High Fat Diet Increases Circulating Endocannabinoids Accompanied by Increased Synthesis Enzymes in Adipose Tissue
Source: Front Physiol. 2019 Jan 10;9:1913. doi: 10.3389/fphys.2018.01913 (PMC6335353; doi:10.3389/fphys.2018.01913)
Supplement: Supplementary file 2 [file Data_Sheet_2.zip › Supplementary Figures.docx]

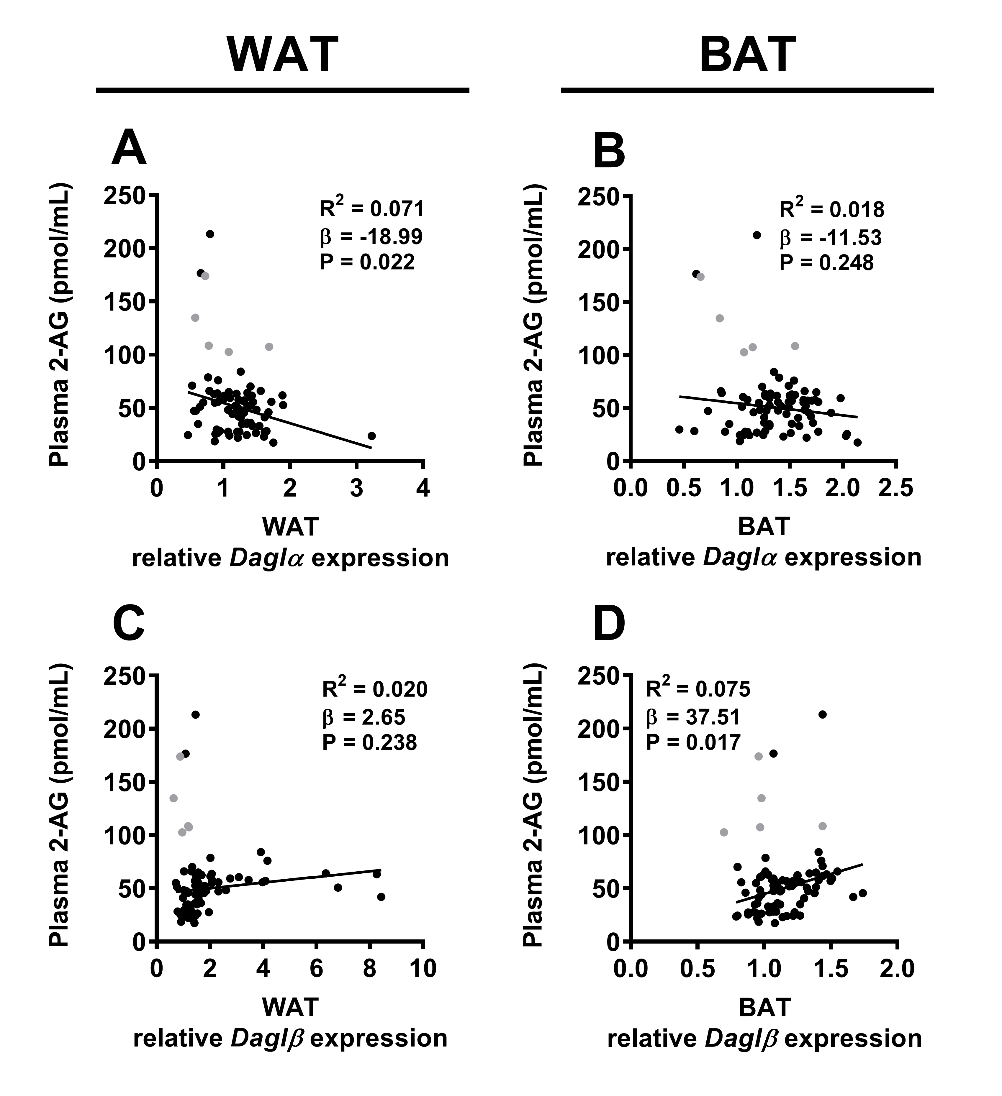


**Supplementary Figure 1**. Linear regression analysis was performed on correlations between DAG lipase expression in the adipose tissues and plasma 2-AG levels. Linear regression analysis for *Daglα* expression relative to 0 weeks of HFD in WAT (**A**) and BAT (**B**) and 2-AG levels and linear regression analysis for *Daglβ* expression relative to 0 weeks of HFD in WAT (**C**) and BAT (**D**) and 2-AG levels. Correlations are shown for all samples depicted in black (n=81). Samples depicted in grey were regarded as biological outliers based on 2-AG and AA levels and therefore excluded from linear regression analyses.

**
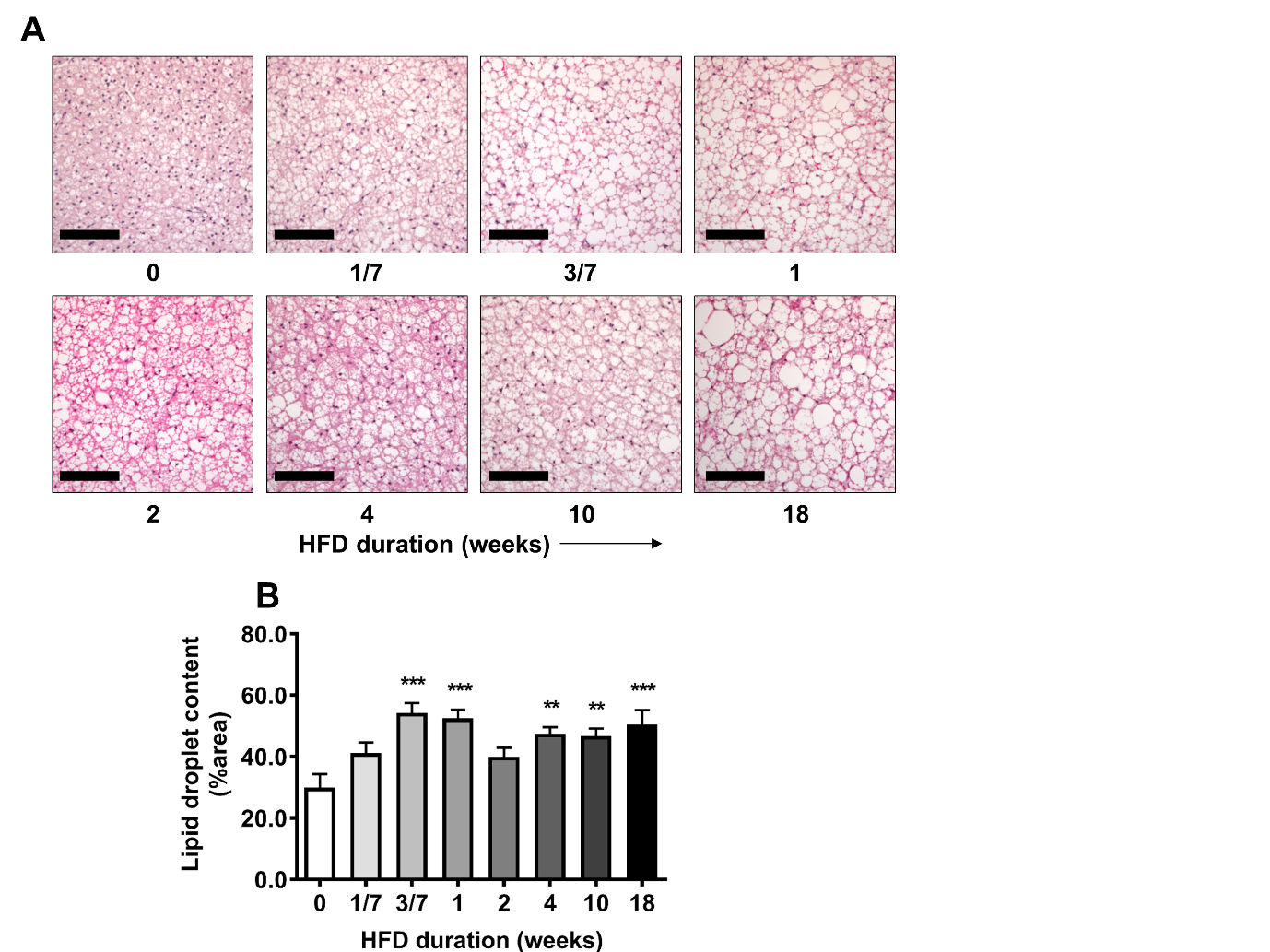
**

**Supplementary Figure 2.** BAT sections were stained for H&E and representative images are shown (**A**). Pictures were analysed in ImageJ to determine the lipid droplet content (**B**). Data are represented as mean ± SEM (n=9-11) ***P*<0.01; ****P*<0.001 compared to the control (0 weeks of HFD) group analysed by one-way ANOVA with Dunnett’s posthoc test. Bars (A) indicate 100 µm.
